# Clinical Anatomy and Significance of the Retromolar Foramina and Their Canals: A Literature Review

**DOI:** 10.7759/cureus.1781

**Published:** 2017-10-17

**Authors:** Mindy K Truong, Puhan He, Nimer Adeeb, Rod J Oskouian, R. Shane Tubbs, Joe Iwanaga

**Affiliations:** 1 Harvard School of Dental Medicine, Harvard University; 2 Department of Neurosurgery, Louisiana State University, Shreveport, LA.; 3 Neurosurgery, Complex Spine, Swedish Neuroscience Institute; 4 Neurosurgery, Seattle Science Foundation; 5 Seattle Science Foundation

**Keywords:** retromolar, canal, foramen, dental implant, anatomy, variation, clinical

## Abstract

The retromolar foramina (RMF) and the retromolar canal (RMC) are anatomic variants in the mandible located distally to the last molar. The retromolar nerve, which runs through the RMC, is a type 1 bifidity of the mandibular canal. The investigations of the RMF and RMC have been performed by dry mandible studies, the panoramic radiograph (PAN), computed tomography (CT), and the cone beam computed tomography (CBCT) studies. The CBCT has been shown to be the superior method for visualizing the RMF and RMC. There is wide variation in the frequency, location, diameter, and distance of the canal in different individuals. Overall, there is no significant difference in the frequency of the canal in the mandible between sexes or sides of the mandible. The peak incidence of the RMF may occur in adolescence. The RMC is significant due to the neurovascular bundle which runs through it. Injury to this neurovascular bundle during surgical procedures, such as third molar extraction, implant placement, or split sagittal osteotomy, may lead to paresthesia, excessive bleeding, or traumatic neuroma. The presence of RMC may also lead to insufficient anesthesia in the mandible which may be overcome with alternative anesthetic techniques.

## Introduction and background

**Figure 1:**
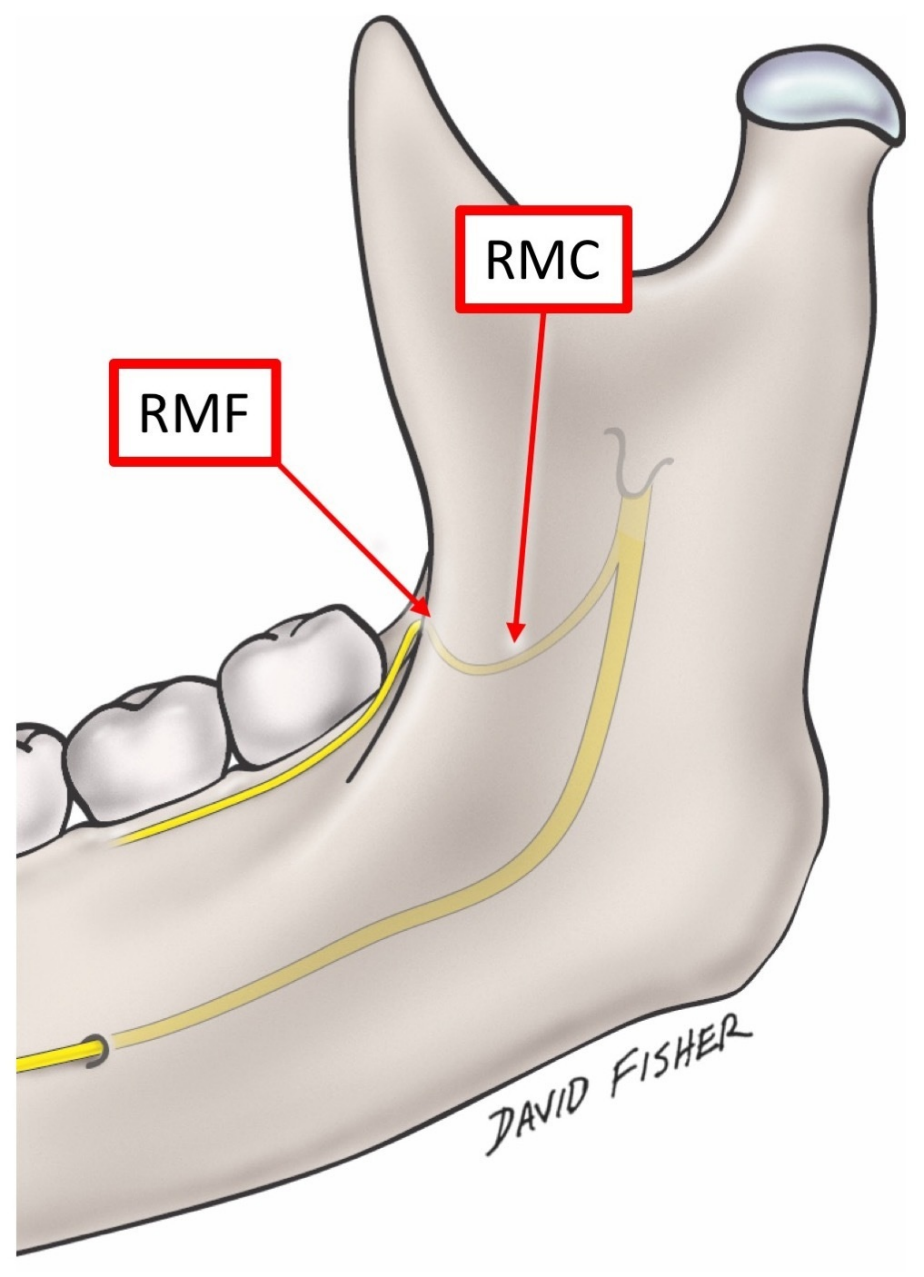
The retromolar foramen, canal and the nerve. RMF: retromolar foramina; RMC: retromolar canal

Newer imaging modalities have brought attention to accessory foramina of the mandible, such as accessory mental foramina (AMF) [1], lingual foramina [2] and retromolar foramina (RMF). Of these, the RMF is known as apertures of the retromolar canal (RMC) in the mandible (Figure 1), which has clinical importance due to the presence of a neurovascular bundle which runs through it [3]. The RMF is located posteriorly to the last molar in the retromolar trigone, which is bounded anteriorly by the third molar, medially by the temporal crest, and laterally by the anterior border of the ramus [4].

The RMC is a type 1 bifidity of the mandibular canal [5]. A bifid mandibular canal (BMC) is an anatomical variation wherein the mandibular canal divides into two parts. Each branch may carry its own neurovascular bundle [6]. It is suggested that bifid and trifid mandibular canals occur due to incomplete fusion of separate mandibular canal nerves from the incisors, primary molars, and permanent molars during embryonic development [7]. The bifidity may be classified by its course. A type 1 bifidity is a unilateral or bilateral transverse bifidity. A type 2 bifidity is unilateral or bilateral and is limited to the ramus or body of the mandible. A type 3 bifidity is a combination of type 1 and type 2, thus it is a transverse and horizontal bifidity [8]. Another variation of the mandibular canal is the double mandibular canal (DMC), in which a second mandibular canal originates from a separate foramen, called the double mandibular foramen (DMF), located near the mandibular foramen [9]. The nerve that runs through the RMC might arise from the early accessory branches of the inferior alveolar nerve (IAN) or long buccal nerve [10]. This area is commonly invaded during mandibular third molar surgery, autologous bone harvesting [11], and sagittal split osteotomy. It is suggested that the RMC may provide accessory innervation to the third mandibular molars from the mandibular canal. It is also possible that the RMC, particularly types 2 and 3, contains aberrant buccal nerves which penetrate the buccinator muscle [10].

The most common variation of the RMC is a branch of the mandibular canal below the third molar. The nerve travels in a posterosuperior direction and opens in the retromolar fossa those posterior to the third molar [12]. The second variation of the RMC opens in an anterior direction and the branches of the IAN as it enters the mandibular canal. The third and rarer variation of the RMC splits from a more proximal branch of the mandibular canal and enters the bone through a canal at the temporal crest, exiting anteriorly through the retromolar foramen [13].

This review will highlight the clinical importance of RMF and RMCs. An evaluation of the current technologies available to visualize these structures will be presented. Next, this review will characterize RMF by frequency, location, diameter, and distance. Finally, clinical implications and pathologies associated with these structures will be presented, as well as suggestions for further research.

## Review

Technology

The most common technologies used to detect RMF and RMCs are cone beam computed tomography (CBCT), computed tomography (CT), and the panoramic radiography (PAN), with CBCT being the most sensitive technique [14]. Interestingly, one study used endoscopy to observe the mandibular foramen, which clearly demonstrated the mandibular canal branching of the RMC [15].

Cone Beam Computed Tomography Versus Panoramic Radiography

Motamedi, et al. [8] reported a prevalence of the RMC, or type I BMC, detected by PAN of less than 1%, while CBCT studies have detected a much higher incidence. Han and Park, et al. [16] reported visualization of the RMC on the sagittal and cross-sectional images of two Korean patients using CBCT, however, PAN failed to visualize the same structures. Therefore, CBCT can be useful in confirming anatomical variations of the mandibular canal which cannot be visualized on PAN [10].

Muinelo-Lorenzo, et al. [17] examined the presence and morphology of BMCs and RMF in 225 subjects using the CBCT and PAN. The BMCs were detected on CBCT in 83 out of 225 patients (36.8%). Of these, the PAN visualized only 37.8% of the BMCs and 32.5% of the RMF was seen on CBCT. The study concluded that PAN is insufficient in identifying BMCs and RMF, and will likely lead to an underestimation of these structures. The CBCT should be considered superior to PAN in the determination of these anatomic structures. Von Arx, et al. [18] found similar results in his study, in which 31 RMCs in total were identified on CBCT in 121 sides (25.6%), and only seven of these 31 RMCs (23%) were detected in the corresponding PAN. Both studies suggested that PAN may have a lower detection rate due to the small diameter of the RMC. Sisman, et al. [19] conducted a study in which 947 hemimandibles in 632 patients were examined. In total, 253 RMCs (144 left and 109 right) were identified on CBCT imaging (26.7%), while only 3.06% were detected on PAN.

Disadvantages of the Cone Beam Computed Tomography Imaging

One disadvantage of CBCT includes the possible presence of artifacts, defined as discrepancies between the reconstructed visual image and the actual subject, which degrades the quality of these images. Furthermore, structures that do not exist in the actual subject may appear in the image due to the patient motion, image capturing, and the process of reconstruction. The CT artifacts can include noise, motion, beam hardening, scatter, and metal artifacts [20].

Disadvantages of the Panoramic Radiography

One disadvantage of PAN includes ghost shadows which are produced by the contralateral side of the mandible, pharyngeal airway, soft palate, and uvula, which may impede the detection of the mandibular canal. The PAN has been reported to be unable to detect buccally and lingually bifurcated canals [21]. Furthermore, the PAN may be less sensitive in detecting thin canals and foramina, such as RMCs and AMF, compared to CBCT imaging [22]. Other disadvantages include lack of detail, irregular magnification, geometric distortion, and overlapping of the anatomical structures [23]. Iwanaga, et al. [24] reported multiple accessory foramina of the mandibular ramus, which were suspected by the PAN and confirmed using the CT.

Cone Beam Computed Tomography Versus Computed Tomography

According to Naitoh, et al. [25] in a study comparing CT and CBCT imaging in 28 patients, four out of 19 of the BMCs detected on the CBCT were not visualized on the CT. Thus, CBCT showed a higher incidence of bifurcation of the mandibular canal than CT. However, the voxel size used in the settings for the CT was 0.5 mm, while the voxel size used for the CBCT was 0.2 mm. This difference in voxel size could affect the detection rate of anatomic structures. Furthermore, artifacts caused by metal crowns or fillings could influence the detection of anatomic variants of the mandibular canal in the retromolar area. The CBCT has advantages over the CT, including (1) relatively lower radiation dose, (2) more affordable equipment and (3) higher image quality of bone tissue [26].

Panoramic Radiography Versus Spiral Computed Tomography Versus Limited Cone Beam Computed Tomography

Fukami, et al. [27] compared bilateral BMCs of a Japanese cadaver visualized by the PAN, spiral CT, and limited CBCT. Cross-sectional limited CBCT images of the canals were compared to gross anatomical sections of the mandible and were found to be consistent. The spiral CT and limited CBCT images showed the bilateral BMCs, while PAN only showed the left BMC. Furthermore, the distribution of the canal was more distinctive in the images of the limited CBCT than in the spiral CT. Thus, limited CBCT is a valuable imaging technique to assess the distribution of BMCs.

Most Valuable Visualization Technology: Cone Beam Computed Tomography

In conclusion, CBCT is the best imaging technique for identifying the RMC and can be used when a preliminary radiograph fails to delineate a clear relationship between the IAN and other anatomical structures in the mandibular molar area [14]. Limited CBCT may be extremely valuable for assessing the presence of BMCs. It is clinically important to localize a BMC, such as the RMC, prior to dentoalveolar surgery. This is especially significant when the presence of BMCs is suspected by PAN [27].

Anatomical variations in the retromolar foramina

Frequency of the Retromolar Foramina

**Figure 2:**
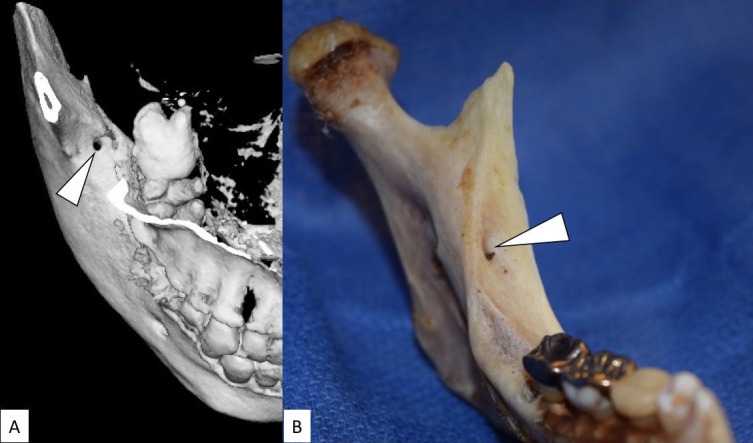
The retromolar foramen. The retromolar foramen (arrowhead) A: displays the right side in the computed tomography (superior view) B: displays the left side in the dry mandible (anterior-medial view).

The frequency of RMF as reported by the CBCT studies ranges from 5.4% [6] and 75.4% [28]. The frequency of RMF reported by human dry mandible studies ranges from 3.2% [12] to 72% [29] (Figure 2). The frequency of RMF as reported by the PAN studies ranges from 3.06% [19] to 8.8% [23] (Tables 1-2). This large range can be attributed to several factors, including ethnic differences, environmental and genetic factors, and variation in sample sizes across studies [26]. However, studies have suggested that RMF and RMCs are normal anatomical variations of the IAN, rather than anomalies [5].

**Table 1:** The frequency, side, diameter, and the distance of the retromolar foramen in various populations based on cone beam computed tomography and panoramic studies. Symbols: - not related, * no gender predilection, ** no side predilection.

Authors	n	Population	Freq	Sex	Left Side	Right Side	Unilateral	Bilateral	Mean Diameter	Boundaries	Mean Distance
*Cone beam computed tomography studies*											
Filo, et al. [30]	680	Swiss	16.1%	*	42.53%	33.3%	75.9%	24.14%	1.03 mm (0.4-2.0)	To distal cementoenamel junction of second molar	15.10 mm (2.7-24.8)
Han and Hwang [10]	446	Korea	8.5%	*	23.7%	57.9%	81.6%	18.4%	1.13 mm (±0.38, 0.60–2.00)	To second molar	14.08 mm (±3.85, 8.50–24.00)
Kang, et al. [6]	1933	Korea	5.4%	*	-	-	-	-	1.36 mm (±0.60, 0.27-3.29)	Unspecified	16.20 mm (±4.67)
Kawai, et al. [31]	46	Japanese	37%	-	-	-	-	-	-	-	-
Lizio, et al. [14]	187	Italian	16%	-	-	-	-	-	-	-	-
Naitoh, et al. [25]	122	Japanese	25.4%	-	-	-	-	-		Mean length of canal	14.8 mm
Ogawa, et al. [32]	319	Japanese	28%	-	-	-	92%	8%	0.9 (±0.4, 0.2-3.2)	Distance to third molar	5.5 mm (±2.1, 1.7-11.1)
Orhan, et al. [33]	242	Turkish	28.10%	*	-	-	-	-	-	Mean length of canal	13.5 mm
Patil, et al. [28]	171	Japanese	75.4%	*	-	-	56.5%	44.5%	-	-	-
Rashsuren, et al. [34]	500	Korean	17.4%	*	-	-	-	-	2.2 mm (±0.5)	-	-
Sisman, et al. [19]	632	Turkish	26.7%	*	-	-	-	-	-	-	-
von Arx, et al. [18]	100	Swiss	25.6%	*	**	-	81%	19%	0.99 mm (±0.31)	To second molar	15.16 mm (±2.39, 12.32-22.32)
Panoramic studies											
Capote, et al. [23]	500	Brazilian	8.8%	*	29.5%	47.7%	77.3%	22.7%	-	-	-
Sisman, et al. [19]	632	Turkish	3.06%	-	-	-	-	-	-	-	-
von Arx, et al. [18]	100	Swiss	5.8%	-	**	-	-	-	-	-	-

**Table 2:** The frequency, side, diameter, and the distance of the retromolar foramen in various populations based on human dry mandible studies. Symbols: - not related, * no gender predilection, ** no side predilection, ***reported mean diameter of 1.7 mm (1.1–2.1).

Authors	n	Population	Freq	Sex	Left Side	Right Side	Unilateral	Bilateral	Boundaries	Mean Distance
Alves, et al. [3]	22	Black	27%	*	33.3%	33.3%	66.6%	33.3%	-	-
	64	White	15.6%	*	50%	20%	70%	30%	-	-
Bilecenoglu, et al. [35]	40	Turkish	25%	-	**	-	75%	20%	To second molar distal edge	11.91 mm (±6.71)
	-	-	-	-	-	-	-	-	To third molar distal edge	4.23 mm (±2.30)
Gamieldien, et al. [36]	885	South African	8%	*	-	-	-	-	To second molar distal edge	16.8 mm (±5.6)
Hosapatna, et al. [37]	50	South Indian	6.0%	-	-	-	-	-	-	-
Kodera, et al. [38]	41	Japanese	19.5%	-	-	-	-	-	-	-
Motamedi, et al. [39]***	136	-	40.4%	*	-	-	43.6%	56.4%	From lingual cortex	-
Narayana, et al. [40]	242	Indian	21.9%	-	32.1%	49%	81.1%	18.9%	-	-
Ossenberg [12]	86	Italian	8.1%	*	-	-	-	-	-	-
	94	Japanese	3.2%	-	-	-	-	-	-	-
	485	Eskimos	40%	-	-	-	-	-	-	-
	11	Canadians	9.1%	-	-	-	-	-	-	-
Potu, et al. [4]	94	Indian	11.7%	-	27.3%	45.4%	72.7%	27.3%	To posterior border of third molar socket	6.21 mm (±2.01, 4-11)
	-	-	-	-	-	-	-	-	To anterior border of ramus	6.57 mm (±2.82, 3-11)
	-	-	-	-	-	-	-	-	To lingula	4.43 mm (±1.87, 2-8)
Priya, et al. [41]	157	Indian	17.8%	-	39.3%	32.1%	71.4%	28.6%	-	-
Pyle, et al. [42]	249	African American	7.8%	*	-	-	-	-	-	-
	226	Caucasian	8.4%	*	-	-	-	-	-	-
Sagne, et al. [43]	99	Swiss	20.2%	*	-	-	-	-	-	-
Sawyer, et al. [13]	234	American	7.7%	*	**	-	94.4%	-	-	-
Scheijtman, et al. [29]	18	Argentine aborigines	72%	-	-	-	73%	27%	To third molar distal edge	10.5 mm (±3.8)

Locations of the Retromolar Foramina

The RMF is found in the retromolar fossa above the occlusal plane and below the coronoid process of the ramus [42]. The retromolar area is bounded by the external oblique ridge, the attachment of the pterygomandibular raphe and the last molar in the mandible [36]. The histological analysis determined that the retromolar nerve extends from the anterior border of the ramus and continues to the buccal gingival of up to two teeth anteriorly in the first molar region [5]. Potu, et al. [4] found that RMF is located mostly in the medial aspect of the retromolar fossa, proximal to the lingula.

According to Haas, et al. [26], the RMF on the right side of the mandible was overall found to be positioned further distally in the retromolar region than on the left side of the mandible. Some cases, particularly in RMCs with a large diameter (> 1 mm), were positioned more anteriorly. Rarely, in cases with large diameters, the RMF was positioned in the anterior temporal crest of the coronoid process.

Diameter of the Retromolar Foramina

The diameter of RMF has been reported to range from 0.2 mm [32] to 3.29 mm [6] (Tables 1-2). Males have been reported to have larger diameters of RMF, which can be explained by the fact that male mandibles are usually larger than in females [5].

Distance/Length of the Retromolar Foramina

The reported distances between RMF and the distal edge of the third molar were between 4.23 mm [35] and 10.5 mm [29]. The reported distances between the RMF and the distal edge of the second molar were between 11.91 mm [35] and 16.8 mm [36] (Tables 1-2). These values suggest that the locations of RMF are not constant.

Age

Capote, et al. [23] found no significant difference in the presence of RMF based on age. According to Ossenberg [12], the peak incidence of RMF occurs in the adolescent cohort. This may reflect the increased neurovascular requirements in adolescents for the growth, spurt and the eruption of the third molars. Furthermore, the preferential distribution of the nerve that runs through the RMC on the temporalis tendon may relate to the adolescent peak of RMF and increased masticatory strength.

Sex

Eighteen of the 29 cited studies found no gender predilection for the presence of RMF, also none of the studies found a gender predilection (Tables 1-2).

Laterality and Number

Capote, et al. [23] found a significant right-sided lateralization of the RMF (p < 0.05; Fisher’s Exact Test) (Table 1). Seven of the 33 cited studies present percentages of RMF in the right and left sides of the mandible. Four studies demonstrate right-sided prevalence [4, 10, 23, 40], two studies demonstrate left-sided prevalence [30, 41] and one study with two separate populations demonstrates equal-sided prevalence and left-sided prevalence [3] (Tables 1-2). Three studies cite no side predilection [13, 18, 35]. Gamieldien, et al. [36] stated that the side in which the foramen presents is not likely to have any developmental, surgical, or anatomical significance. Alves, et al. [3] reported one case of bilateral double RMF and one case of double left RMF. He, et al. reported a tripled RMF [44].

Clinical relevance

Contents of the Retromolar Canal 

The RMC originates from the mandibular canal, follows a recurrent path, and ends in either RMF or in nearby foramen. The contents of the RMC are derived from their inferior dental homologues and include a myelinated nerve, one or more arterioles, and one or more venules [29]. According to Bilecenoglu, et al. [35], an artery is present in a lumen of 120 to 130 mm. After departing the body of the mandible, these entities distribute mainly upon the temporalis tendon, buccinator muscle, the most posterior zone of the alveolar process, and the mandibular third molar [29]. The distal end of the RMC extends to the distal root of the third molar and retromolar area, demonstrating that the contents of the RMC provide innervation and vascular supply for the third molar and mucosa of the retromolar area [35].

Insufficient Anesthesia

**Figure 3:**
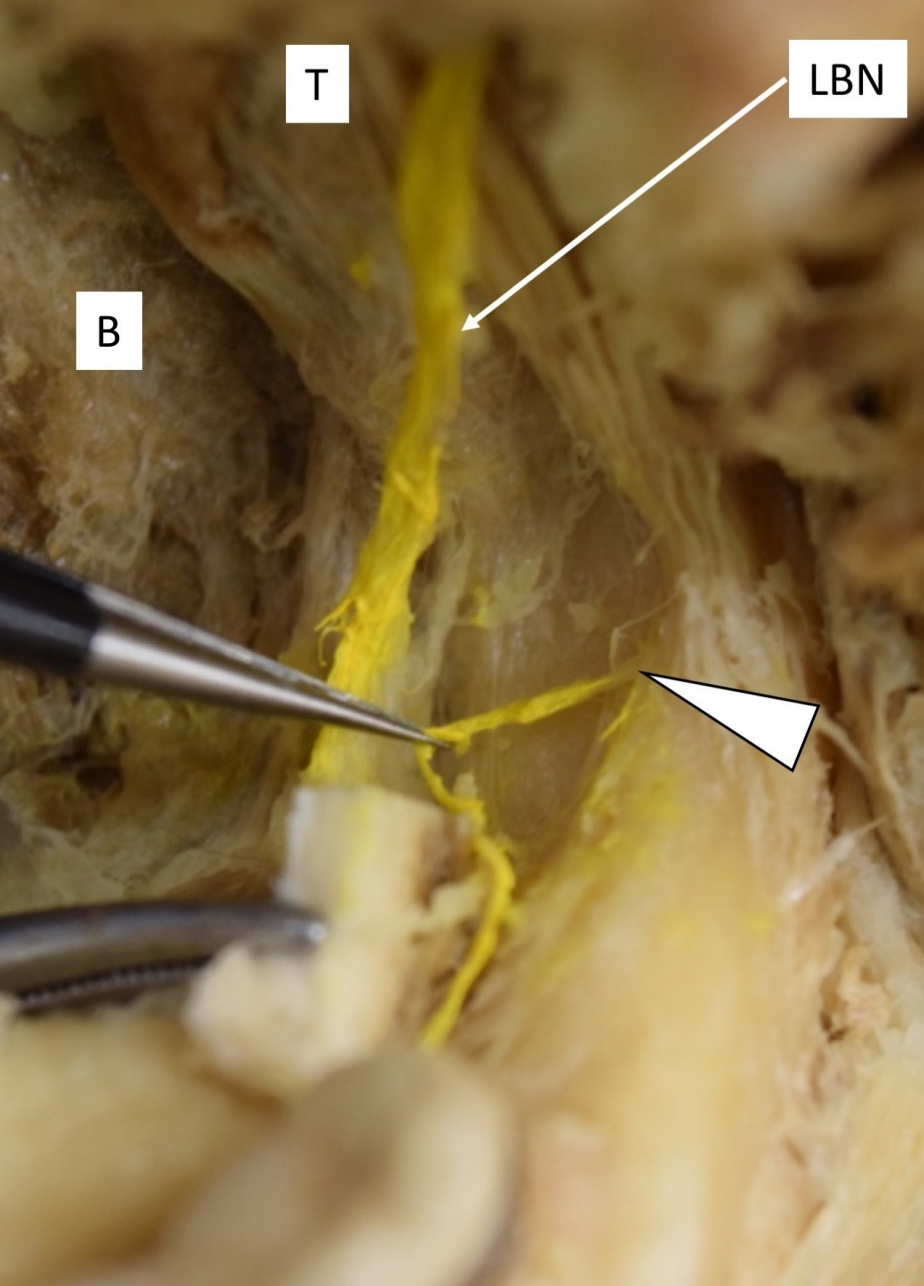
The medial view of the right mandible. Note: The retromolar nerve arose from the retromolar foramen (arrowhead) and is distributed to the mandibular molar buccal gingiva B: buccinator muscle, LBN: long buccal nerve, T: temporalis.

The nerve fibers which branch from the mandibular canal and exit through RMF may prevent complete anesthesia of the mandibular buccal gingiva [35] (Figure 3). The nerves which exit from RMF may innervate the temporal tendon, buccinator muscle, posterior portion of the mandible, third molars, gingiva of the mandibular molars and premolars, and the mucosa of the retromolar pad [29, 38].

The direct, also known as the standard technique of IAN block is the most widely used approach, however, it does not always achieve complete anesthesia. The potential causes of IAN block failure have been discussed extensively, some of which is the position of the mandibular foramen [45], innervation of the lingual cortical plate by the nerve to the mylohyoid [46], and central core theory [47]. The presence of the RMC is one of the possible causes of incomplete anesthesia of the mandibular molars despite IAN block anesthesia. In the event of incomplete anesthesia due to the presence of the RMC, a few anesthetic drops injected into the retromolar region may achieve the desired result [30]. In identified cases involving bifid canals, it may be advisable to perform a higher anesthetic technique, such as the Gow-Gates technique. The Gow-Gates technique anesthetizes all mandibular nerve branches following anesthetic injection at a single point in the pterygomandibular space, including any nerves of the RMC. Another alternative to achieve full anesthesia in the event of BMCs with the failure of the conventional IAN block is the Akinosi-Vazirani technique. The Akinosi-Vazirani technique occurs with the patient’s mouth closed and the local anesthesia is injected to fill the pterygomandibular space. Both the Gow-Gates and Akinosi-Vazirani techniques are indicated for any procedures to be performed in the mandibular arch and are especially useful when the patient had a history of standard IAN block failure due to anatomic variation or accessory innervation, such as in the presence of the RMC [42, 48].

Surgical Procedure Complications

Anatomical variants such as the RMC, if unidentified, may lead to complications when performing intraoral procedures such as third molar extraction, autologous bone harvesting [11], or sagittal split ramus osteotomy. The most common complications include paresthesia, traumatic neuroma, bleeding, hematoma, or bruising [48].

Excessive Bleeding

The artery in the RMC bifurcates into facial and buccal branches after exiting the retromolar foramen. Injury to this artery in the perimandibular retromolar region during surgery may lead to excessive bleeding in the presence of BMCs and RMF [27]. However, to our knowledge, there have not been any reports of excessive bleeding due to injuring the RMF.

Autologous Bone Graft

For the purpose of oral surgery procedures, which usually only require small amounts of bone material, there may be a preference towards intraoral donor sites. Compared to extraoral sites, intraoral sites offer the advantages of easier surgical access, reduced surgery time, lack of cutaneous scars, and reduced morbidity. The disadvantage of the intraoral site is the limited amount of bone, which may be harvested. The two most important intraoral donor sites include the retromolar region and the symphysis menti. Several studies have correlated the retromolar region with less postoperative morbidity compared to the symphysis menti, which suggests that the retromolar region is the “first choice” donor site. Retromolar autologous bone withdrawal is also associated with the lower risk of complication compared to branch sagittal osteotomy or osteogenic distraction technique. However, it is essential to have a thorough understanding of the posterior mandibular region in order to limit the possibility of injuring nerves and vasculature in the area, including the mandibular canal [11, 49].

Mucoperiosteal Flap Elevation

If the presence of RMF is not detected prior to mucoperiosteal flap elevation, damage may occur to the neurovascular contents of the RMC, leading to paresthesia of the areas supplied by the retromolar nerve. The greater the area supplied by the retromolar nerve, the greater the risk of injury and loss of sensation [12].

Prosthetics and Dental Implant Placement

Prosthetic restorations, such as dentures or dental implants, placed distally to the retromolar area may impinge on the contents of the RMC and lead to discomfort, pain or paresthesia. This may be especially significant in the elderly due to alveolar bone resorption [28].

Spread of Infection or Tumors

The vasculature of the RMC is a possible route for the spread of infection or tumors from the oropharynx to systemic circulation [4].

Muscle Innervation

The retromolar nerve provides innervation to the buccinator and temporalis muscles, and damage to this nerve may disrupt the function of these muscles [4].

Clinical Case

Singh, et al. [50] reported a case of permanent paresthesia of the buccal mucosa in the area from the retromolar region to the canine on the operated side after injuring a branch of the buccal nerve which crossed the retromolar foramen during the extraction of a third molar.

## Conclusions

The results of this study suggest that RMF and RMCs have wide anatomical variation among individuals. It is important to locate anatomic variants of RMF and RMCs before performing the surgical procedures in the retromolar area and to achieve full anesthesia.

While several studies concluded that the CBCT imaging technology is the most valuable and accurate method of visualizing retromolar foramina and canals, there is no established protocol for determining when it is appropriate to utilize the CBCT from the evaluation of a radiograph or the PAN. It would be useful to have established guidelines that allow dental clinicians to determine what imaging features or clinical characteristics suggest follow-up with the CBCT for further evaluation of the presence of RMF and RMCs.
